# Enteric neuronal cell therapy reverses architectural changes in a novel diphtheria toxin-mediated model of colonic aganglionosis

**DOI:** 10.1038/s41598-019-55128-4

**Published:** 2019-12-10

**Authors:** Sukhada Bhave, Emily Arciero, Corey Baker, Wing Lam Ho, Rhian Stavely, Allan M. Goldstein, Ryo Hotta

**Affiliations:** Department of Pediatric Surgery, Massachusetts General Hospital, Harvard Medical School, Boston, MA USA

**Keywords:** Enteric nervous system, Enteric neuropathies, Animal disease models

## Abstract

Hirschsprung disease (HSCR) is characterized by absence of the enteric nervous system (ENS) in the distal bowel. Despite removal of the aganglionic segment, gastrointestinal (GI) problems persist. Cell therapy offers potential treatment but use of genetic models is limited by their poor survival. We have developed a novel model of aganglionosis in which enteric neural crest-derived cells (ENCDCs) express diphtheria toxin (DT) receptor. Local DT injection into the colon wall results in focal, specific, and sustained ENS ablation without altering GI transit or colonic contractility, allowing improved survival over other aganglionosis models. Focal ENS ablation leads to increased smooth muscle and mucosal thickness, and localized inflammation. Transplantation of ENCDCs into this region leads to engraftment, migration, and differentiation of enteric neurons and glial cells, with restoration of normal architecture of the colonic epithelium and muscle, reduction in inflammation, and improved survival.

## Introduction

The enteric nervous system (ENS), which consists of neurons and glia arranged in networks of ganglia within the wall of the gastrointestinal (GI) tract, regulates multiple critical aspects of gut function, including motility. The ENS arises from neural crest-derived cells that migrate along the length of the developing GI tract. Enteric neuropathies, which are characterized by the congenital absence, acquired loss, or abnormal function of intrinsic enteric neurons, represent severe clinical GI disorders for which effective treatments are lacking because they do not address the underlying pathophysiology^1,2^. One potential therapy is enteric neuronal cell transplantation^3^.

One of the best understood enteric neuropathies is Hirschsprung disease (HSCR), a congenital disorder in which enteric ganglia are absent along a variable length of the distal intestine due to failure of enteric neural crest-derived cells (ENCDCs) to complete their colonization of the intestine during development. The uncolonized colon remains aganglionic and tonically contracted, resulting in functional obstruction and impaired GI motility. The incidence of HSCR is 1 in 5000 live births, of which 80% have short-segment HSCR, in which only the rectosigmoid colon lacks ganglion cells^4,5^. Current treatment for HSCR involves surgical removal of the aganglionic segment, but the functional outcome is variable and often accompanied by long-term GI problems, including enterocolitis, constipation, fecal incontinence, and reduced quality of life. New and more effective therapies for the treatment of HSCR are needed^3,6,7^.

Several genetic models of intestinal aganglionosis are commonly used to explore the potential of cell therapy as a novel treatment for HSCR^8–10^. These include rodent models with mutations in genes essential for ENS development, such as members of the *Ret* tyrosine kinase receptor pathway^11–13^, the transcription factor *Sox10*^14,15^, or the *Et3*-*Ednrb* signaling pathway^16–19^. Each of these models, however, has limitations for long-term studies since the animals die within the first several weeks of life due either to bowel obstruction or development of enterocolitis. The short lifespan prevents long-term follow-up after cell therapy. A chemically induced model of aganglionosis, which relies on benzalkonium chloride (BAC), is hampered by off-target effects, with non-specific ablation of smooth muscle cells and interstitial cells of Cajal^20,21^. Furthermore, reinnervation of the gut has been observed just seven days following BAC treatment^22^.

To allow studies on cell therapy for aganglionosis, we have developed a novel mouse model of focal intestinal aganglionosis by targeting ENCDCs. We generated a transgenic model in which diphtheria toxin receptor (DTR) is expressed on neural crest-derived cells, leaving them susceptible to the toxic effect of diphtheria toxin (DT). Focal injection of DT into the colon reliably produces segmental aganglionosis. Importantly, this model does not rely on a specific genetic mutation and exhibits improved survival compared to the genetic models, because the aganglionosis is focal (non-circumferential) and does not adversely affect gut motility. We have successfully transplanted ENCDCs into this novel model of colonic aganglionosis and observed their survival, migration, and differentiation. Importantly, we found that ENCDCs restored normal colonic architecture that was perturbed by ENS ablation. The longer survival rate of this model will provide a valuable platform to test cell-based therapy as an innovative treatment for intestinal aganglionosis. Furthermore, this model will be useful for assessing ENS interactions with other cell types, including intestinal epithelial cells, immune cells, endothelial cells and the luminal microbiome, allowing studies on how the ENS influences motility, secretion, barrier integrity, microvascular circulation, and local immunity^23–25^.

## Results

**Figure 1:**
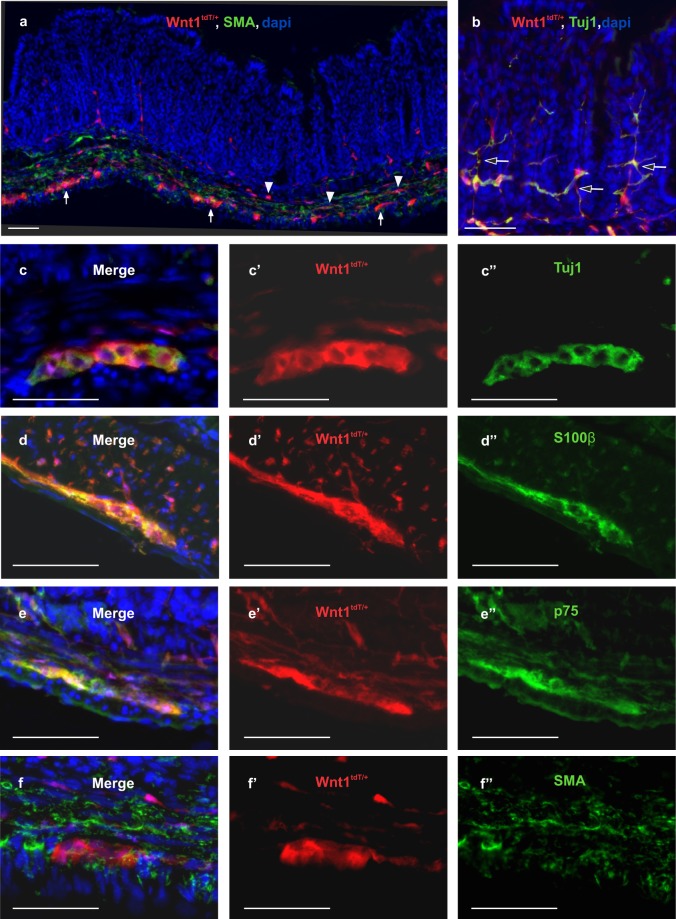
Characterization of colonic ENS in Wnt1^tdT/+^ mice. Colon of Wnt1^tdT/+^ mice expresses tdT in the submucosal (a, arrowheads) and myenteric plexi (**a**, arrows). tdT + fibers are found projecting to the mucosa (**b**, open arrows). Cross sections of Wnt1^tdT/+^ colon show tdT colocalizing with Tuj1 (**c-**c”), S100β (**d-**d”), and p75 (**e**-e”), but not with SMA (**f**-f”). Scale bar 50 µm (**a–e**).

We have established a novel model of segmental intestinal aganglionosis using transgenic expression of DTR in neural crest-derived cells followed by administration of DT. In this model, ENS ablation was achieved by utilizing *Wnt1*^*Cre*/+^ mice. To characterize Wnt1 expression in mouse intestine, *Wnt1*^*Cre*/+^ mice were first crossed with *R26R-tdTomato (R26R-tdT)* reporter mice to generate a *Wnt1*^*tdT*/+^ line. Cross sections from the colon of *Wnt1*^*tdT*/+^ mice showed expression of tdT in the myenteric plexus (Fig. 1a, closed arrows) and submucosal plexus (Fig. 1a, arrowheads), with tdT fibers projecting into the mucosa (Fig. 1b, open arrows). In myenteric ganglia, tdT expression colocalized with neuronal marker, Tuj1 (Fig. 1c–c”), glial marker, S100β (Fig. 1d–d”), and neural crest cell marker, p75 (Fig. 1e–e”), but not with smooth muscle marker, SMA (Fig. 1f–f”).

**Figure 2:**
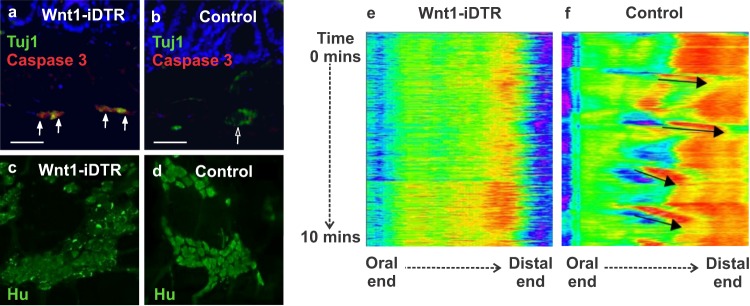
Systemic administration of DT to Wnt1-iDTR mice leads to enteric neuronal loss and colonic dysmotility. Intraperitoneal delivery of DT causes enteric neuronal apoptosis in the colon of Wnt1-iDTR mice (**a**, arrows) but not iDTR-negative controls (**b**). This is confirmed by wholemount immunostaining showing disrupted Hu-expression in Wnt1-iDTR mice (**c,d**). Spatiotemporal mapping of colonic contractility shows absence of CMMCs in DT-treated Wnt1-iDTR mice (**e**), as compared to normal contractile activity in DT-treated control mice (f, arrows). Scale bar 50 µm (**a,b**).

*Wnt1*^*Cre*/+^ mice were crossed with *R26R-iDTR* reporter mice to generate Wnt1-iDTR transgenic mice, in which DTR is selectively expressed in neural crest-derived cells. Active Cre recombination in these mice renders Wnt1-expressing cells sensitive to DT. As a proof of concept, to achieve ENS ablation, DT (40 µg/kg) was administered via intraperitoneal (i.p.) injection into Wnt1-iDTR mice and littermate controls (n = 3 mice per group). Within 2 days after DT administration, cleaved caspase-3 expression increased in the colonic myenteric ganglia of Wnt1-iDTR mice (Fig. 2a, arrows) as not in DT-treated, Cre-negative controls (Fig. 2b). Furthermore, the myenteric plexus of the colon of Wnt1-iDTR mice showed disrupted Hu-immunoreactivity as compared to the control mice (Fig. 2c,d). The effect of ENS ablation on colonic motility was assessed by spatiotemporal mapping. Colonic migrating motor complexes (CMMCs) were absent in Wnt1-iDTR mice (Fig. 2e), while control mice exhibited normal coordinated contraction patterns (Fig. 2f, black arrows) after DT injection. These results demonstrate successful *in vivo* ablation of neural crest-derived cells following DT administration to Wnt1-iDTR mice and associated colonic dysmotility in this novel model of intestinal aganglionosis.

**Figure 3:**
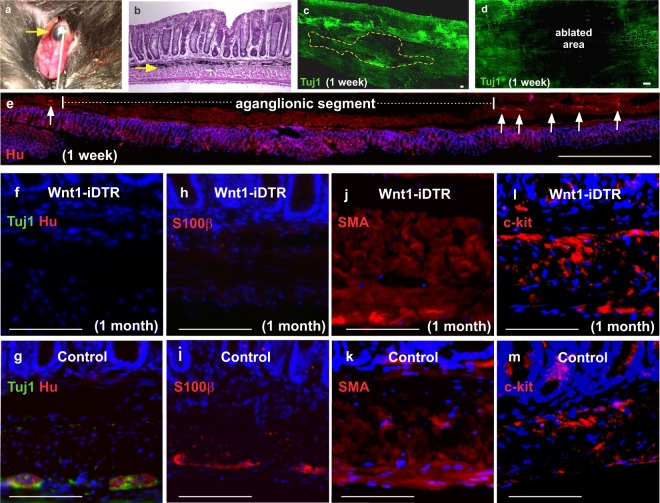
Intracolonic injection of DT results in focal aganglionosis. DT was injected into the mid-colon via laparotomy (**a**). Injection site is marked by India ink (**a,b** arrows). Immunofluorescence one week later shows focal loss of myenteric neurons (ablated area marked by a dotted line; **c**) by wholemount staining for Tuj1 (**c,d**) and Hu staining of a longitudinal section of mid-colon (**e**, arrows mark endogenous neurons at margins of DT ablation). One month after DT injection, focal ablation of Tuj1 + and Hu + enteric neurons (**f,g**) and S100β + enteric glia **(h,i**) was observed, with no obvious loss of SMA + smooth muscle (**j,k**) or c-kit + interstitial cells of Cajal (l,m). Scale bar 100 µm (**c,d**), 1 mm (**e**), and 100 µm (**f–m**).

While ENS ablation was achieved following i.p. DT administration, 100% of these mice die 2 days following injection due to expression of DTR in all cells derived from the neural crest lineage. Therefore, to create a non-lethal model of intestinal aganglionosis, DT injection was targeted specifically into the gut wall to limit neural crest cell injury to a focal region of intestine. DT was injected into the wall of the mid-colon of Wnt1-iDTR (n = 14 mice) and control (n = 9 mice) animals via laparotomy (Fig. 3a). India ink was added to the DT to mark the injection site (Fig. 3a,b, arrow). Based on testing of multiple concentrations of DT to achieve ENS ablation without adversely impacting survival, we chose a single injection of 4 ng (4 µl of 1 ng/µl) DT. While 2 ng DT did not ablate enteric neural crest-derived cells (ENCDCs), an 8 ng DT dose produced ENS ablation but also resulted in a megacolon phenotype and mortality at about five days. Wholemount longitudinal muscle myenteric plexus (LMMP) immunostaining showed that a single injection of 4 µl of 1 ng/µl DT ablated the ENS in an area spanning 25 ± 2 mm^2^ of the myenteric plexus at 1 week after DT injection, with preservation of enteric neurons surrounding the injection site (Fig. 3c; ablated area marked by a dotted line, 3d). Longitudinal sections showed a 4–6 mm aganglionic segment in the colonic wall one week after DT injection, as confirmed by the absence of Hu expression in the myenteric plexus (Fig. 3e, arrows point to enteric neurons). Enteric neuronal and glial loss persisted at one month following DT injection in Wnt1-iDTR mice (Fig. 3f–i), with aganglionosis persisting up to 3 months following injection (Supplementary Fig. 1). Smooth muscle cells (Fig. 3j,k) and interstitial cells of Cajal (Fig. 3l,m) were preserved, verifying that ablation is specific to the ENS. Following focal ENS ablation, the five-week survival was 71.4 ± 12.1% (n = 14), with the remainder dying with functional intestinal obstruction and a megacolon phenotype.

**Figure 4:**
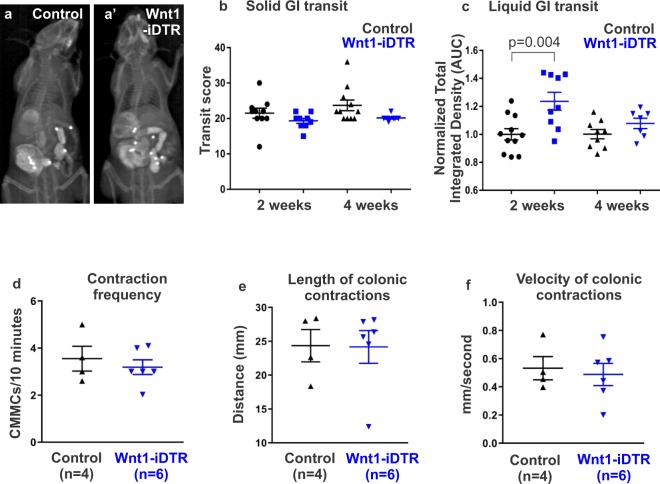
DT-induced focal aganglionosis does not affect total GI transit and colonic motility. A radiologic-based transit assay (**a**,a’) was used to measure solid and liquid transit following DT injection. No difference was observed in solid transit at 2 and 4 weeks after DT injection (**b**). Liquid transit showed a delay at 2 weeks, but not at 4 weeks (**c**). *Ex vivo* analysis by spatiotemporal mapping of the DT-injected colon from Wnt1-iDTR mice showed no changes in CMMC frequency (**d**), length (**e**), or velocity (**f**), as compared to controls.

To determine whether focal ENS ablation affects gut motility, we utilized *in vivo* radiologic imaging to measure solid and liquid whole GI transit. This method allows longitudinal analysis of GI transit over time without the need to sacrifice the animals at each time point. Figures 4a,a’ show representative x-ray images taken from control and Wnt1-iDTR mice, respectively, 2 weeks after DT injection. No difference was observed in solid transit at 2 weeks (21.5 ± 1.4 in controls, n = 10, vs 19.3 ± 0.7 in Wnt1-iDTR, n = 9) or 4 weeks following DT (23.7 ± 1.5 in controls, n = 11, vs 20.1 ± 0.3 in Wnt1-iDTR, n = 7) (Fig. 4b). For liquid transit, the amount of barium left in the GI tract was 20% greater in Wnt1-iDTR mice at 2 weeks (p = 0.004), suggesting slower liquid transit compared to controls. However, no difference was observed between the two groups at 4 weeks (Fig. 4c).

Colonic motility following focal ENS ablation was examined by spatiotemporal mapping four weeks after DT injection. D-maps were generated to quantify the frequency, length, and velocity of CMMCs in control versus Wnt1-iDTR mice. No significant difference was seen in the frequency (3.4 ± 0.5 CMMCs in controls, n = 4, vs 3.3 ± 0.3 CMMCs in Wnt1-iDTR, n = 6, Fig. 4d), length (24.5 ± 1.7 mm in controls, n = 4, vs 24.8 ± 1.1 mm in Wnt1-iDTR, n = 6, Fig. 4e) or velocity (0.51 ± 0.049 mm/s in controls, n = 4, vs 0.5 ± 0.045 mm/s in Wnt1-iDTR, n = 6, Fig. 4f) of CMMCs. These findings suggest that, unlike systemic DT administration, focal DT-mediated ENS ablation did not significantly perturb GI motility.

**Figure 5:**
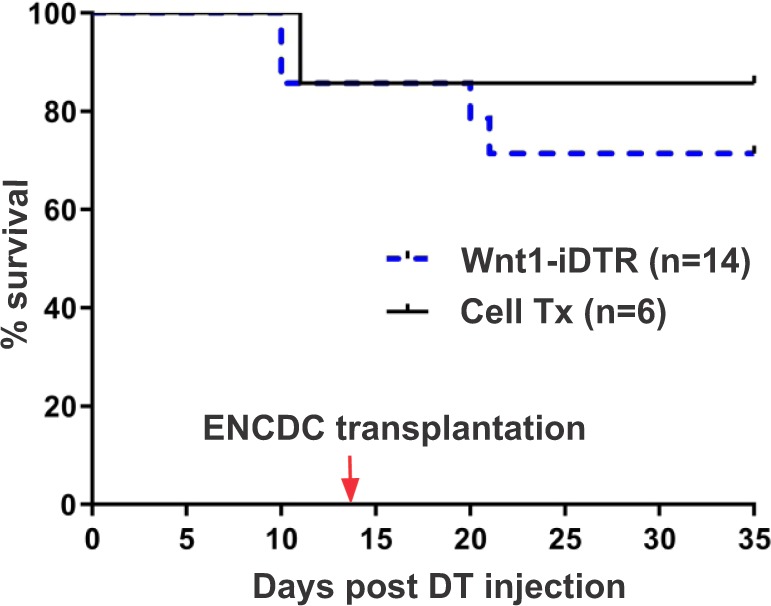
ENCDC transplantation improves survival following ENS ablation in Wnt1-iDTR mice. Kaplan-Meier survival curves show that ENCDC transplantation results in an improved five-week survival rate in mice where ENS ablation was induced by DT injection.

**Figure 6:**
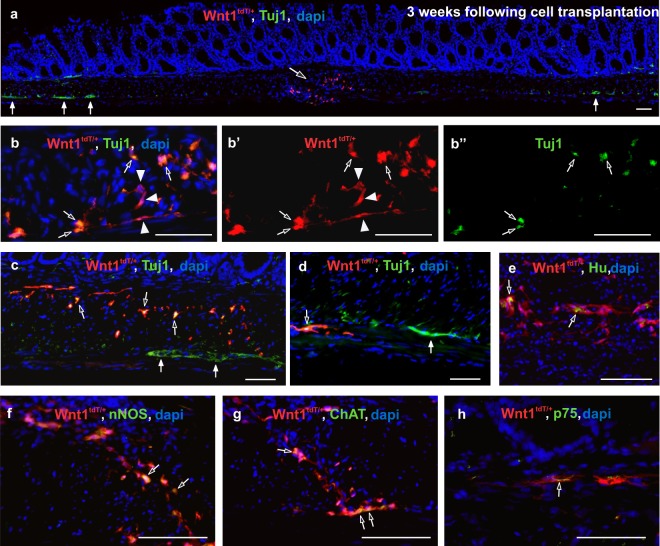
Transplanted ENCDCs survive, engraft, and migrate in the aganglionic colon. Two weeks after DT injection, tdT + neurospheres were transplanted into the colon wall and analysis was performed at 3 weeks after cell transplantation. Transplanted tdT + neurospheres (**a**, open arrow) survive in the aganglionic segment of the colon (**a**, closed arrows mark endogenous ganglia). Transplanted cells consist of Tuj1 + neurons (**b-**b”, open arrows) and Tuj1-negative neural crest-derived cells (**b**-b’, arrowheads). Transplanted tdT + cells (c, open arrows) are found near endogenous ganglia (**c**, closed arrows) and project fibers within the muscle layers (**d**, open arrow; closed arrow marks endogenous myenteric plexus. Wnt1-tdT ENCDCs express Tuj1 (**b,c,d)**, Hu (**e**), nNOS (**f**), ChAT (**g**), and p75 (**h**). Scale bar 50 µm (**a**–**h**).

We used this novel model of focal colonic aganglionosis, free of dysmotility or poor survival, as a platform to study neuronal stem cell therapy. tdT + ENCDCs were isolated from the small intestinal LMMP of 3-week old Wnt1^tdT/+^ mice and cultured to form neurospheres^26^. ENS ablation was induced with DT injection into the mid-colon of Wnt1-iDTR mice as described above. Two weeks later, Wnt1^tdT/+^ enteric neural crest-derived neurospheres were injected into the colon wall of recipient mice at the DT injection site. Recipient colons were examined three weeks after neurosphere transplantation. 14.29% mice died within the first two weeks after DT injection. ENCDC transplantation resulted in 85.7 ± 13.2% overall survival (n = 6 mice), as compared to 70% survival without cell therapy (Fig. 5). Transplanted tdT + neurospheres (Fig. 6a, open arrow) were identified within the aganglionic segment, which was flanked by endogenous Tuj1 + ganglia (Fig. 6a, closed arrows). Higher magnification images show that tdT + cells migrated out of the neurospheres, extended fibers, and spread along the intermyenteric layer of the gut wall (Fig. 6b). Transplanted cells covered 1.2 ± 0.7 mm length of the aganglionic colon segment (n = 5). Transplanted cells consisted of neurons (Fig. 6b–b”, open arrows) and non-neuronal cells (Fig. 6b–b”, arrowheads). tdT + cells (Fig. 6c,d, open arrows) were found adjacent to the endogenous ganglia (Fig. 6c,d, closed arrows). Transplanted cells also express Hu (Fig. 6e), nNOS (neuronal nitric oxide synthase) (Fig. 6f), ChAT (choline acetyl transferase) (Fig. 6g), and p75 (Fig. 6h). The findings suggest that the DT-induced aganglionic gut environment is permissive to transplanted ENCDCs.

**Figure 7:**
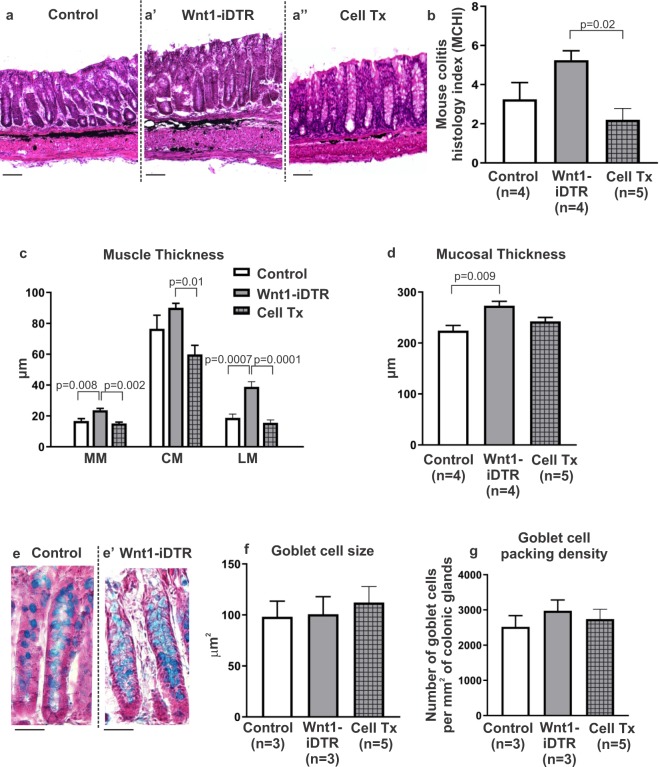
ENCDC transplantation reverses the effects of DT-induced aganglionosis on colonic architecture. Representative images are shown of H&E stained colon 4–5 weeks following DT injection into control (**a**), non-transplanted Wnt1-iDTR (a’), and ENCDC-transplanted Wnt1-iDTR (a”) mice. MCHI scores show low-grade inflammation in all three groups (**b**), with transplanted colon showing significantly less inflammation. DT-mediated ENS ablation leads to increased thickness of muscularis mucosa (MM) and longitudinal muscle (LM), with no significant change in circular muscle (CM), and these changes are reversed following cell transplantation (**c**). DT-mediated ENS ablation also increases mucosal thickness, which is partially normalized by ENCDC transplantation (**d**). Alcian blue staining (**e**,e’) shows no change in goblet cell size **(f**) or density (**g**) among the groups. Scale bar 100 µm (**a**-a”) and 50 µm (**e**,e’).

Although focal ENS ablation did not affect motility, architectural changes were observed in the colon. To characterize these changes, and determine how neuronal stem cell therapy affects them, colon sections were examined 4–5 weeks after DT injection. Alterations to the mucosal architecture were common in the setting of DT-induced aganglionosis despite DT being injected only into the muscle layers. These alterations included elongation of the crypts, a reduction in crypt density, or inconsistent crypt widths, whereas the columnar brush border appeared unaffected. Low-grade inflammation was detected in H&E-stained sections in both control and Wnt1-iDTR mice following DT injection with mouse colitis histology index (MCHI) scores ranging from 1 to 6, out of a possible maximum score of 22 (3.25 ± 0.85 in controls, n = 4, vs 5.25 ± 0.48 in Wnt1-iDTR, n = 4, Fig. 7a,a’,b). ENCDC transplantation into the ablated region of Wnt1-iDTR mice significantly improved MCHI score (2.2 ± 0.58, n = 5, p = 0.02, Fig. 7a”,b) compared to Wnt1-iDTR mice that did not undergo cell transplantation.

ENS ablation was also associated with a significant increase in thickness of the muscularis mucosa (23.7 ± 1.2 µm in Wnt1-iDTR, n = 4, vs 16.8 ± 1.5 µm in controls, n = 4, p = 0.008) and longitudinal muscle (38.9 ± 3.3 µm in Wnt1-iDTR, n = 4, vs 18.8 ± 2.4 µm in controls, n = 4, p = 0.0007) (Fig. 7c). ENCDC transplantation reversed this in both the muscularis mucosa (15.24 ± 0.8 µm, n = 5, p = 0.002) and longitudinal muscle (15.66 ± 1.8 µm in Cell Tx, n = 5, p = 0.0001) (Fig. 7c). Interestingly, circular muscle thickness was increased, though not significantly, following ENS ablation (90 ± 2.9 µm in Wnt1-iDTR, n = 4, vs 76.6 ± 8.7 µm in controls, n = 4, Fig. 7c). ENCDC transplantation significantly reduced circular muscle thickness in Wnt-iDTR mice (59.9 ± 5.9 µm, n = 5, p = 0.01).

Mucosal thickness was also markedly increased following ENS ablation (273.1 ± 8.7 µm in Wnt1-iDTR, n = 4, vs 224 ± 10.1 µm in controls, n = 4, p = 0.009) (Fig. 7d). Consistent with this, analysis of crypt proliferation using Ki67 demonstrated a marked expansion of the proliferative zone within the ENS ablated area (Supplementary Fig. 2a, injection marked by dye) as compared to regions distant from the ablated site (Supplementary Fig. 2b). ENCDC transplantation to the ablated segment reduced the thickness of the mucosa though this did not reach statistical significance (242.7 ± 7.7 µm, n = 5, p = 0.07, Fig. 7d).

Finally, Alcian blue staining was performed to quantify goblet cell size and packing density (Fig. 7e,e’). ENS ablation did not affect goblet cell size (100.7 ± 17.3 µm^2^ in Wnt1-iDTR, n = 3. vs 98.2 ± 15.4 µm^2^ in controls, n = 3, Fig. 7f), nor did ENCDC transplantation (112.2 ± 15.7 µm^2^, n = 5, Fig. 7f). Since mucosal thickness was different between the two groups, we analyzed the number of goblet cells per area of colonic glands. There was no difference in goblet cell packing density (goblet cell number per mm^2^ of colonic glands) between Wnt1-iDTR and control mice following DT injection (2977 ± 306.3 goblet cells/mm^2^ in Wnt1-iDTR mice, n = 3. vs 2517 ± 323.1 goblet cells/mm^2^ in controls, n = 3, Fig. 7g), and ENCDC transplantation did not alter this parameter (2737 ± 280.8 µm^2^, n = 5, Fig. 7g).

## Discussion

Abnormalities of the ENS comprise a group of GI motility disorders that include HSCR, esophageal achalasia, intestinal pseudo-obstruction, and gastroparesis. Developing neuronal stem cell-based therapies to replace missing or abnormal enteric neurons has gained momentum recently, especially for the treatment of HSCR^3,6,27–29^. However, the utility of genetic rodent models of HSCR for long-term studies is hindered by their short lifespan, typically 4 weeks. An alternative approach to creating segmental aganglionosis relies on topical application of a detergent, BAC. However, BAC has several drawbacks, including nonspecific injury to non-ENS cells, ablation only of the myenteric plexus, and reinnervation of the region beginning about one week later^30–32^. The limitations of existing genetic and experimental models of aganglionosis hamper the ability of investigators to study the effects of new therapeutic strategies, including stem cell therapy.

We developed a novel animal model of aganglionosis in which lineage-specific ablation of neural crest-derived cells is achieved by administration of DT. Use of Cre-inducible human DTR as a means of cell ablation was first described for ablation of T and B cells^33^, and subsequently used to model human diseases^34–36^. As initial proof of concept, we administered DT systemically to Wnt1-iDTR mice and demonstrated ablation of enteric ganglion cells with resulting functional dysmotility. However, systemic administration of the toxin ablated all neural crest-derived cells, leading to mortality within 48 hours. DT-inducible ablation of PLP1-expressing enteric glial cells was recently described, but the animals exhibited hindlimb weakness, coordination defects, and death by 2 weeks due to expression of PLP1 in peripheral Schwann cells and oligodendrocytes^37^. Therefore, to avoid off-target injury, we specifically ablated enteric neurons and glia within a defined segment of gut by local injection of DT directly into the intestinal wall.

Our model of experimentally-induced focal aganglionosis has several advantages. First, ablation is limited to enteric neurons and glia, making it a more specific model of aganglionosis. This model can therefore be used to study the effects of ENS ablation on other intestinal cell types, as we have done. Second, unlike with BAC treatment, both myenteric and submucosal plexuses are ablated, and the aganglionosis in our model persisted, without re-innervation, for at least three months after DT injection. The absence of re-innervation might be due to the greater extent of aganglionosis achieved by ablating both plexi, as opposed to BAC treatment which only targets the myenteric plexus, as the submucosal plexus might provide progenitors capable of repopulating the myenteric ganglia. Third, this model is associated with improved long-term survival (70% of mice survived at least five weeks after ablation) as compared to genetic HSCR models. The 30% mortality that was observed due to functional obstruction may be due to technical variability in the depth of DT injection and spread of the toxin into the gut wall. Importantly, early deaths within the first few days did not occur, suggesting absence of systemic spread of DT following focal injection. Finally, the extent of aganglionosis, and therefore the severity of the colonic phenotype, can be controlled by varying the volume and area of DT injection. At a higher dose of 8 ng DT (4 µl of 2 ng/µl), a megacolon phenotype was observed with significant dysmotility and death at around 5 days. In contrast, a 4 ng DT (4 µl of 1 ng/µl) dose produced a smaller area of ablation and preserved motility and survival. This ability to modulate the effect is a strength of the system. It is not surprising that a non-circumferential patch of aganglionosis has no impact on motility. For example, in the Duhamel operation for HSCR, a patch of aganglionic rectum is retained and anastomosed side-to-side to the normally ganglionated pull-through segment^38^. In this procedure, half of the circumference of the rectum is aganglionic, but colorectal motility is maintained. To summarize, our model of focal colonic aganglionosis is highly specific, consistent, and devoid of spontaneous reinnervation and limited survival.

We found that segemental aganglionosis did not result in dysmotility, and this is advantageous as it avoids the development of functional intestinal obstruction and subsequent lethality. One exception to this is that we did observe a delay in liquid transit at 2 weeks following DT injection. Interestingly, this resolved at 4 weeks, suggesting the existence of compensatory mechanisms capable of restoring GI transit despite absence of reinnervation of the aganglionic segment. Identifying those mechanisms could have important therapeutic value for improving motility in neurointestinal diseases. We did observe significant morphological changes in gut structure resulting from focal ENS loss. In the ENS ablated segment of the colon, we found a statistically significant increase in the thickness of the muscularis mucosa and longitudinal muscle. Jejunal muscularis hypertrophy was previously reported in a BAC-induced ENS ablation model^39^. Similar observations have been made in an endothelin receptor B (Ednrb)-null rat, a model for HSCR, where increased thickness of the smooth muscle layer was observed in the aganglionic region^40^. We also observed other changes in epithelial architecture, including an increase in mucosal thickness. Increased small intestinal villus length has been reported following BAC-induced ENS ablation^41,42^. Similarly, surgical denervation of the myenteric plexus by stripping the serosa and longitudinal muscle from the intestinal wall leads to an increase in villus height and crypt depth^43^. Although the exact mechanism is unknown, previous studies have demonstrated that the ENS contributes to regulation of epithelial stem cell fate and maintenance of intestinal barrier function by release of trophic factors^44^. We speculate that these compensatory adaptations of the mucosa may serve to restore normal absorption following ENS ablation.

This DT-induced model of focal enteric aganglionosis can be leveraged to explore neuronal stem cell therapy for the treatment of intestinal aganglionosis and also to study interactions between the ENS and other cell types within the gut wall. To date, neuronal stem cell transplantation studies in HSCR models have been restricted to 1–2 weeks duration due to the poor survival of these animals, which has been a significant limitation in the field^45–47^. While our model has distinct advantages over existing mouse models of HSCR, it is important to note that our DT model does not represent a true etiological model of the disease since, unlike HSCR, the aganglionosis in our model is not congenital. Therefore, the microenvironment in our DT model likely does not mimic that seen in HSCR and this may have implications for cell-based therapy. The prolonged survival of our focal ENS ablation model allows long-term analysis of the fate of transplanted cells and their ability to reverse intestinal changes caused by aganglionosis. Following ENCDC transplantation into the ablated region of colon, we observed survival of the transplanted cells for at least three weeks and their migration out of the implanted neurospheres and into the aganglionic segment. Transplanted ENCDCs differentiated into neurons and glial cells. Interestingly, cell transplantation into the ENS ablated area was associated with a 85% survival at five weeks, as compared to the 70% survival seen in ablated but not transplanted mice. Importantly, we found that ENCDC transplantation reversed the increase in muscle and mucosal thickness associated with ENS ablation, and also significantly reduced the inflammation seen in the ablated region, thus restoring normal colonic architecture. In summary, we have established a new model of colonic aganglionosis which does not impact gut motility or survival. Utilizing this model, we demonstrate the feasibility of neuronal cell therapy for the treatment of neurointestinal disease.

## Methods and Materials

### Animals

All the animal protocols were conducted in accordance with the procedures reviewed and approved by the Institutional Animal Care and Use Committee at Massachusetts General Hospital. The following mice were obtained from Jackson Laboratory (Bar Harbor, ME, USA): *Wnt1*^*Cre*/+^ mice (*Tg(Wnt1*^*Cre*^*)11Rth Tg(Wnt1-GAL4)11Rth/J*, Stock #003829 and B6.Cg-Tg(Wnt1-Cre)11Rth/MileJ, Stock #009107), Cre-inducible DTR reporter (*R26R-iDTR*) mice (C57BL/6-*Gt(ROSA)26Sor*^*tm1(HBEGF)Awai*^/J, Stock #007900), and *R26R-tdT* reporter mice (B6.Cg-*Gt(ROSA)26Sor*^*tm14(CAG-tdTomato)Hze*^/J, Stock #007914).

*Wnt1*^*Cre*/+^ mice were crossed with *R26R-tdT* reporter mice to generate *Wnt1-Cre*^+^*;R26-tdT* mice (annotated as Wnt1^tdT/+^). *Wnt1*^*Cre*/+^ mice were also crossed with *R26R-iDTR* reporter mice to obtain *Wnt1-Cre*^+^*;R26-iDTR* mice (annotated as Wnt1-iDTR) and *Wnt1-Cre*^*−*^*;R26-iDTR* wild-type littermates (annotated as control).

DNA for genotyping was prepared from toe biopsies from 10 days old pups using REExtract-N-Amp Tissue PCR kit (Sigma Aldrich). Presence of different alleles was tested by PCR using the following primers: for Cre (200 bp product) Forward 5′-ATTGCTGTCACTTGGTCGTGGC-3′ and Reverse 5′-GGAAAATGCTTCTGTCCGTTTGC-3′; for tdT (200 and 300 bp product) Mutant Forward 5′-CTGTTCCTGTA-3′, Mutant Reverse 5′-GGCATTAAAGC-3′, WT Forward 5′-AAGGGAGCTGC-3′ and WT Reverse 5′-CCGAAAATCTG-3′; and for DTR (500 bp product) Forward 5′-GCCACCATGAA-3′ and Reverse 5′-TCAGTGGGAAT-3′.

### Tissue preparation and immunohistochemistry

Tissue preparation and immunohistochemistry were performed as previously described^26^. Cells, whole mount preparations of LMMP and full-thickness gut samples were fixed in 4% paraformaldehyde. For cryosections, full-thickness gut samples were incubated in 15% sucrose at 4 °C overnight, and then in 15% sucrose containing 7.5% gelatin at 37 °C for 1 hour and rapidly frozen at −50 °C. Frozen sections were cut at 12 μm thickness with a Leica CM3050 S cryostat (Leica, Buffalo Grove, IL). For paraffin sections, fixed tissue samples were processed with an Auto Tissue Processor for 12and 8um thick sections were collected on glass slides. Samples were permeabilized with 0.1% Triton X-100 and blocked with 10% donkey serum for 30 minutes. Primary antibodies were diluted in 10% donkey serum and included mouse anti-neuronal class III β-tubulin (Tuj1; 1:400; Covance, Dedham, MA), human anti-Hu (Anna1, 1:16000, kindly gifted by Lennon lab), rabbit anti-p75 neurotrophin receptor (p75; 1:50; Promega, Madison, WI), rabbit anti-S100β calcium-binding protein B (S100β; 1:100; Neomarkers, Fremont, CA), rabbit anti-smooth muscle actin (SMA; 1:100; Abcam, Cambridge, MA), rabbit anti-cleaved caspase-3 (1:250, Cell Signaling Technology), rabbit anti-c-kit (CD117, 1:100, Dako Cytomation), rat anti-Ki-67 (1:50; Biolegend, San Diego, CA), rabbit anti-neuronal nitric oxide synthase (nNOS; 1:200, Thermo Fisher), and goat anti- choline acetyl transferase (ChAT; 1:500, Millipore). Secondary antibodies included donkey anti-rabbit IgG (1:500; Alexa Fluor 488 and 546; Fisher Scientific Life Technologies), donkey anti-goat IgG (1:500; Alexa Fluor 488; Fisher Scientific Life Technologies), and donkey anti-human IgG (1:200, Alexa Fluor 488 and 647; Fisher Scientific Life Technologies). Cell nuclei were stained with DAPI (Vector Labs, Burlingame, CA) and mounted with aqua-poly/mount (Fisher Scientific Polysciences Inc). Images were taken using a Nikon Eclipse 80i microscope, a Nikon A1R laser scanning confocal microscope (Nikon Instruments, Melville, NY) or a Keyence BZX-700 All-In-One Microscopy (Keyence America Itasca, IL).

### DT injection

Diphtheria toxin (Sigma Aldrich, St. Louise, MO, USA, #D0564) was diluted to 1 μg/ml with saline. Mid-colon was exposed following laparotomy under general anesthesia using isoflurane inhalation and a single dose of DT was injected into the wall of the colon (4 μl of 1 μg/ml DT diluted in India ink) of 3-month-old Wnt1-iDTR and control mice. For systemic administration, we injected 40 μg/kg DT intraperitoneally (i.p.).

### Isolation and culture of ENCDCs

ENCDCs for cell transplantation were isolated from the small intestinal LMMP layer of *Wnt1*^*tdT*/+^ mice. After the mice were euthanized, the small intestine was collected and placed in ice-cold PBS. LMMP was separated from the underlying submucosa using fine forceps, then finely minced into 1 mm pieces and enzymatically dissociated with dispase (250 μg/mL; StemCell Technologies, Vancouver, BC) and collagenase XI (1 mg/mL; Sigma Aldrich, St. Louis, MO) at 37 °C for 40 minutes. Single cells were isolated by filtration through a 40 μm filter and plated at 50,000 cells/mL in a 25-cm^2^ flask in mouse proliferation media, consisting of Neurocult Mouse Basal Medium (StemCell Technologies) supplemented with 10% Neurocult Mouse Proliferation Supplement (StemCell Technologies), 20 ng/mL epidermal growth factor (StemCell Technologies), 10 ng/mL basic fibroblast growth factor (StemCell Technologies), 0.0002% Heparin (StemCell Technologies) and antibiotics (penicillin and streptomycin). After 7 days, primary cell aggregates (neurospheres) were obtained. Neurospheres from *Wnt1*^*tdT*/+^ mice were used for cell transplantation.

### Transplantation of ENCDCs to distal colon *in vivo*

Focal colonic aganglionosis was created by injecting 4 μl of 1 μg/ml DT diluted in India ink into the wall of mid-colon in 3-month-old *Wnt1-iDTR* mice as described earlier. Cell transplantation was performed 2 weeks later. Recipient mice were anesthetized by isoflurane inhalation. Mid-colon was exposed following laparotomy. A 10 μl suspension containing ~500 Wnt1-tdT neurospheres (60–90 µm diameter) in 18% Pluronic^®^ F-127 (PF-127) thermosensitive hydrogel (Sigma, St Louis MO) were microinjected through a 30 G needle into the wall of the mid-colon where DT had been injected to ablate the ENS beforehand^48^. Recipient mice were sacrificed 3 weeks after cell transplantation and longitudinal sections of the mid-colon were analyzed for cell engraftment. This group is annotated as Cell Tx.

### Histological analysis

Colon longitudinal cryosections were stained with hematoxylin and eosin (H&E). Data was collected from n = 3–5 mice per group, and 5 high power (10–20x) images of muscularis mucosa (MM), circular muscle (CM), longitudinal muscle (LM), and mucosal layer were taken per mouse. Muscle and mucosal thickness were measured using ImageJ software. The mouse colitis histology index (MCHI) was measured according to previously recommended criteria^49^. Briefly, MCHI scoring included the assessment of goblet cell loss (0–3), crypt hyperplasia (0–6), crypt density (0–4), and submucosal infiltrate (0–9), with a score of 22 denoting maximum inflammation. All measurements were made by a blinded assessor using Image J software.

For goblet cell labeling, cryosections of colon tissues were incubated with 3% acetic acid (Fisher Scientific), followed by Alcian blue (Sigma-Aldrich, St. Louis, MO) for 30 minutes at room temperature. After rinsing the sections, Nuclear Fast Red (Vector Labs) was added for 5 minutes at room temperature, and then the slides were rinsed in water followed by 95% and 100% alcohol. To measure goblet cell size and packing density, 10–15 goblet cells per gland were analyzed in a blinded fashion from n = 3–4 mice per group^50^. All measurements were made by a blinded assessor using Image J software.

### Gastrointestinal motility monitor (GIMM)

Contractile movement of isolated colon was analyzed *ex vivo* using GIMM (Med-Associates, St Albans, VT, USA). After euthanizing the mice, their colons were excised immediately and placed in an illuminated organ bath continuously perfused with Krebs solution at 36.5 ± 0.5 °C. The Krebs solution was constantly bubbled with carbogen gas (95% O_2_/5% CO_2_). The entire colon containing natural pellets was anchored with pins at the anal and oral ends. Colonic motility was recorded from each mouse for 30 minutes. Video recordings from each animal were used to generate spatiotemporal maps of colonic contractions. Frequency, length and velocity of colonic migrating motor complexes (CMMCs) were analyzed from the spatiotemporal maps using the GIMM processor plugin (Image J)^51,52^. Measurements were made from three 10-minute recordings per mouse and 4–6 mice were analyzed from each group. Supplementary Movies 1 and 2 are accelerated 16-fold to facilitate viewing.

### Measurement of total GI transit using X-ray

All mice were fasted for 16 hours prior to X-ray but had access to water. Mice were briefly sedated with isoflurane inhalation and were gavaged intragastrically with 10 small steel beads (diameter = 0.81 mm; Bal-tec, Los Angeles, CA, USA) and barium sulfate (E-Z Paque, E-Z-EM Canada, Montreal, QC, Canada, 0.3 ml). Four hours after gavage, mice were lightly anesthetized as above and placed prone on the radiation cassette. Posteroanterior views were taken using a portable X-ray unit (50 kV, 1.2 mAs, ScanX14 Portable Digital Imaging Systems). Image files were visualized using Air Techniques ALLPRO Imaging (New York).

The location of the beads and barium along the GI tract was scored as previously reported with a small modification to the published method^53,54^. To analyze solid GI transit, each bead was given a score based on its location within the GI tract (stomach = 1, intestine proximal to DT injection site = 2, intestine distal to DT injection site = 3, expelled = 4). The scores of individual beads within a single animal were then summed together to give a total transit score for that animal. The maximal possible score was 40 and corresponded to the expulsion of all 10 beads. To analyze liquid GI transit, ImageJ software was used to outline the barium in the gut and integrated density (area under the curve) was obtained as a measure of the amount of barium left in the GI tract. Liquid transit of Wnt1-iDTR mice was normalized to their corresponding littermate controls at 2 and 4 weeks post-DT injection (n = 7–9 mice) and expressed as fold change compared to controls (n = 9–11 mice).

### Statistics

Values are represented as mean ± SEM (standard error of mean). Statistical analysis was performed using Prism 7 (GraphPad software, Inc., La Jolla, CA, USA). Statistical significance between two groups was assessed using Student’s t-test. Statistical significance between three groups was assessed using a One-Way ANOVA test with a Tukey’s post-hoc correction. p-values < 0.05 were regarded as significant.

All authors had access to the study data and reviewed and approved the final manuscript.

## Supplementary information

Supplementary Figures

Supplementary Movie 1.

Supplementary Movie 2.
