# Supplementary material for: Enteric neuronal cell therapy reverses architectural changes in a novel diphtheria toxin-mediated model of colonic aganglionosis
Source: Sci Rep. 2019 Dec 10;9:18756. doi: 10.1038/s41598-019-55128-4 (PMC6904570; doi:10.1038/s41598-019-55128-4)
Supplement: Supplementary file 1 — Supplementary Figures [file 41598_2019_55128_MOESM1_ESM.pdf]

Supplementary information

Title- Enteric neuronal cell therapy reverses architectural changes in a novel diphtheria toxin-mediated model of colonic aganglionosis

Short title- Rescue of diphtheria toxin-mediated intestinal aganglionosis

Authors- Sukhada Bhave, Emily Arciero, Corey Baker, Wing Lam Ho, Rhian Stavely, Allan M. Goldstein, Ryo Hotta*

Legends of supplementary figures

Supplementary Figure 1. Focal ENS ablation is maintained for at least 3 months following DT injection in the colon. Representative images showing loss of Hu immunostaining in colon cross sections of Wnt1-iDTR mice 3 months following DT injection (a). Control colon shows positive Hu staining in the myenteric ganglia (b, arrows).

Scale bar 100 µm (a,b).

Supplementary Figure 2. Focal ENS ablation results in increased crypt cell proliferation. Ki67 staining shows a marked increase in epithelial cell proliferation in Wnt1-iDTR mice following DT injection.

Scale bar 50 µm (a,b).

Supplementary Movie 1. Spatiotemporal mapping of motility in a control colon one month after DT injection.

Supplementary Movie 2. Spatiotemporal mapping of motility in Wnt1-iDTR colon one month after DT injection.

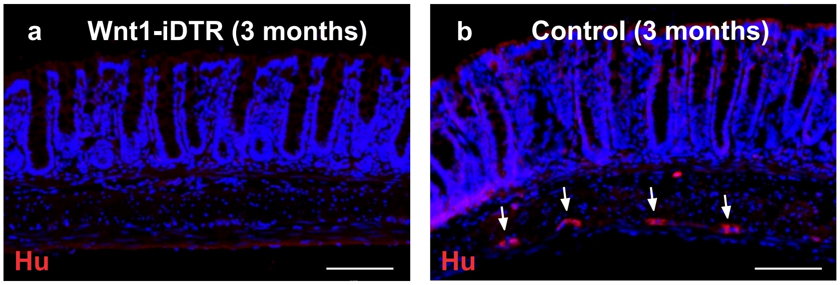
a
Wnt1-iDTR (3 months)
Hu
b
Control (3 months)
Hu

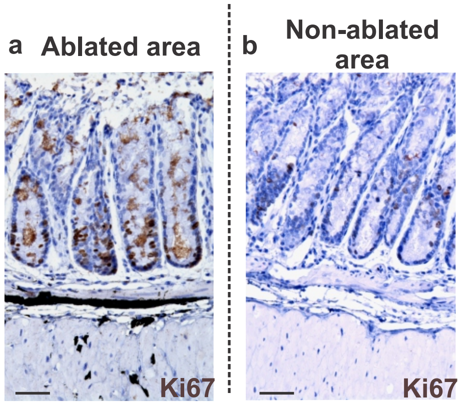
a
Ablated area
Ki67
b
Non-ablated
area
Ki67
